# It Is All About Energy-the Changing Role of X-ray Photons in Photon-counting Detector Computed Tomography: What the Radiologist Needs to Know About kV and keV

**DOI:** 10.1097/RLI.0000000000001283

**Published:** 2026-04-06

**Authors:** Eva J.I. Hoeijmakers, Bibi Martens, Joachim E. Wildberger, Thomas G. Flohr, Cécile R.L.P.N. Jeukens

**Affiliations:** Department of Radiology and Nuclear Medicine, Maastricht University Medical Centre, Maastricht, The Netherlands (E.J.I.H., B.M., J.E.W., T.G.F., C.R.L.P.N.J.); CARIM Cardiovascular Research Institute Maastricht, Maastricht University, Maastricht, The Netherlands (E.J.I.H., J.E.W.); GROW Research Institute for Oncology and Reproduction, Maastricht University, Maastricht, The Netherlands (B.M.); Diagnostic and Interventional Radiology, University Hospital Zurich, University of Zurich, Zurich, Switzerland (T.G.F.)

**Keywords:** computed tomography, image quality, photon-counting CT, kV, monoenergetic CT

## Abstract

CT image quality is strongly influenced by the energy of the x-ray beam. The tube voltage (kV) determines a polyenergetic x-ray spectrum, which affects both image contrast and noise in conventional energy-integrating detector CT (EID-CT) imaging. In photon-counting detector CT (PCD-CT), spectral data sets are always available by default, and virtual monoenergetic images (VMI) can be reconstructed at any specific photon energy (keV). This enables radiologists to tailor energy selection to dedicated clinical tasks, achieving an optimal balance between contrast, noise, and radiation dose, and ultimately enhancing diagnostic confidence. This article educates the reader on the differences between polyenergetic and monoenergetic images to provide radiologists with practical insights into optimizing spectral imaging protocols and leveraging keV differences in routine clinical practice. Using an anthropomorphic phantom with iodine rods, differences in image quality, specifically image contrast and noise, are demonstrated between polyenergetic EID-CT and PCD-CT VMIs.

Computed tomography (CT) has revolutionized diagnostic imaging by providing detailed cross-sectional images of the body. Image quality (IQ) depends heavily on scanner technology, acquisition parameters, patient characteristics, and post-processing algorithms. Importantly, optimal IQ for the given task must be balanced against radiation exposure to minimize the risks associated with ionizing radiation.^1^

CT systems use a broad polyenergetic x-ray spectrum generated by the x-ray tube, with its shape influenced by the tube voltage (kV). In conventional energy-integrating detector CT (EID-CT), the detector sums the energy of all incoming photons, so image contrast and noise are determined by the combined effect of the entire spectrum. EID-CT systems are also capable of spectral imaging through dual-energy CT (DECT) techniques, by acquiring data with 2 different x-ray spectra using techniques such as dual-source systems and rapid kV-switching, or by energy separation on the detector domain with dual-layer detectors.^2^ This leads to the ability to generate virtual monoenergetic images (VMIs), simulating tissue attenuation at a single (monoenergetic) energy level (measured in kiloelectronvolts, keV).^3^

More recently, photon-counting detector CT (PCD-CT) has emerged as a promising next-generation technology that enables spectral imaging as well. This system directly detects individual x-ray photons including their energy, and assigns them to discrete energy bins. PCD-CT offers potential benefits including improved spatial resolution, enhanced contrast-to-noise ratio (CNR), electronic noise removal, and reduced radiation dose.^4^ PCD-CT provides VMIs for default viewing as a standard acquisition mode. Given these advantages, PCD-CT has the potential to become the new standard in clinical CT imaging over the coming decades.^5^

This review educates on the impact of energy on CT IQ, both at the acquisition stage (kV) and during post-acquisition reconstruction using VMIs (keV level). Using an anthropomorphic phantom with iodine rods, polyenergetic EID-CT images and PCD-CT VMIs are compared across various kV and keV levels. Key differences in IQ are demonstrated through noise and contrast graphs. The aim is to provide radiologists with practical insights into optimizing spectral imaging protocols to balance diagnostic IQ with radiation dose.

## COMPUTED TOMOGRAPHY TECHNOLOGY—THE BASICS

### X-ray Tube

An x-ray tube generates x-rays by accelerating electrons from a heated cathode toward a rotating metal anode (typically tungsten) using a high tube voltage (kV). The kV determines the electron acceleration, and thus their energy, for example, at 120 kV, electrons hitting the anode have an energy of 120 keV (Table 1). As the accelerated electrons collide with the anode, they rapidly decelerate, converting most of their energy (>99%) into heat. A small fraction is emitted as x-ray photons, forming a polyenergetic x-ray beam, that is, it contains photons with energies up to 120 keV, called a spectrum. The spectrum is composed of:

1. A continuous Bremsstrahlung spectrum, with photon energies ranging from very low up to the energy determined by the kV value.
2. Discrete characteristic peaks, which are sharp increases in intensity at specific energies, are caused by electron interactions with the atoms of the anode material.

**TABLE 1:** Essential Definitions

keV (kiloelectronvolt): A unit of energy. In CT, keV is used to describe the energy level of individual photons or the reference energy in virtual monoenergetic image reconstructions. kV (kilovoltage): The x-ray tube voltage applied during CT data acquisition. It determines the maximum kinetic energy than an electron can achieve on its way from the cathode to the anode plate of the x-ray tube. For example, at a tube voltage of 120 kV, the maximum electron energy is 120 keV. This is then also the upper energy limit of the polyenergetic x-ray spectrum generated by the incident electrons. At a tube voltage of 120 kV, it includes photons with energies ranging from a few keV up to, but not exceeding, 120 keV. VMI (virtual monochromatic image): A reconstruction generated from spectral data of conventional dual-energy CT or PCD-CT that simulates images as if acquired at a single, specific photon energy level (measured in keV).

Although kV sets the maximum photon energy, most x-ray photons have lower energies.^6^ Therefore, a spectrum is usually characterized not only by its maximum energy, but also by its effective photon energy, which depends on several factors, including beam filtration. Filtration is used to selectively remove low-energy photons at the tube aperture. These photons would be fully absorbed by the patient without contributing to image formation, leading to an unnecessary increase in radiation dose. This pre-patient filtration effectively increases the effective energy of the x-ray beam, producing a “harder” spectrum. Figure 1 shows a typical filtered polyenergetic x-ray spectrum.^7^

**FIGURE 1:**
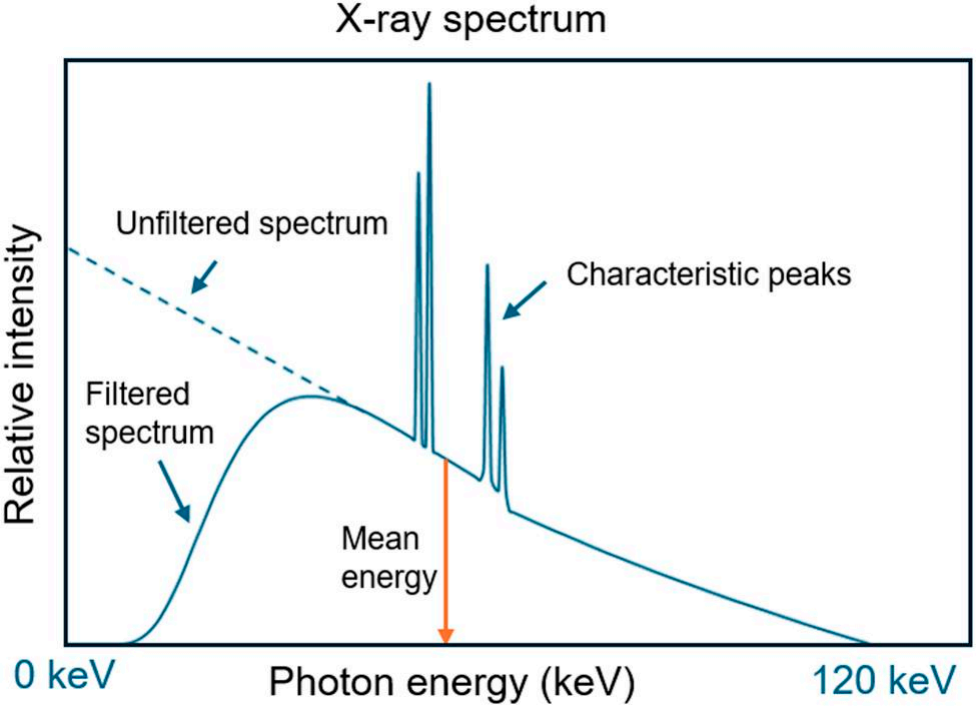
Typical x-ray energy spectrum at 120 kV tube voltage (solid line). The dashed line shows the spectrum without filtration of low-energy photons. The orange arrow indicates the mean energy of the spectrum, which depends on the amount of filtration.^7^

### Image Contrast and Noise in Computed Tomography

The number of x-ray photons reaching the CT detector depends on the total attenuation along their path through the body. Variations in tissue attenuation result in differences in detected photon number and energy, determining image contrast. In CT, attenuation is quantitatively expressed in Hounsfield Units (HU), a standardized scale where water is set at 0 HU and air at -1000 HU. Good contrast enables visualization of subtle density variations, essential for detecting lesions and abnormalities.^8^

X-ray attenuation is primarily caused by 2 physical interactions:

1. Photoelectric effect: The process of photons transferring their full energy to an inner-shell electron, ejecting it from the atom. This process increases with atomic number (Z) of the absorbing material (∝Z³), and decreases sharply with increasing photon energy E (∝1/E³). As a result, it dominates at lower energies (lower kV-spectra) and in high-Z materials such as bone and iodine (Fig. 2A). Hence, iodinated contrast agents are most effective at low kV, where vascular and lesion contrast is maximized.^9^
2. Compton scattering: The process of a photon transferring part of its energy to an outer-shell electron. The photon is deflected with reduced energy, while the electron is ejected from the atom. Because Compton scattering is relatively independent of Z, it produces more uniform attenuation, resulting in less attenuation differences between tissues. It is the dominant interaction at higher energies and in low-Z tissues such as fat and soft tissue (Fig. 2A).

**FIGURE 2:**
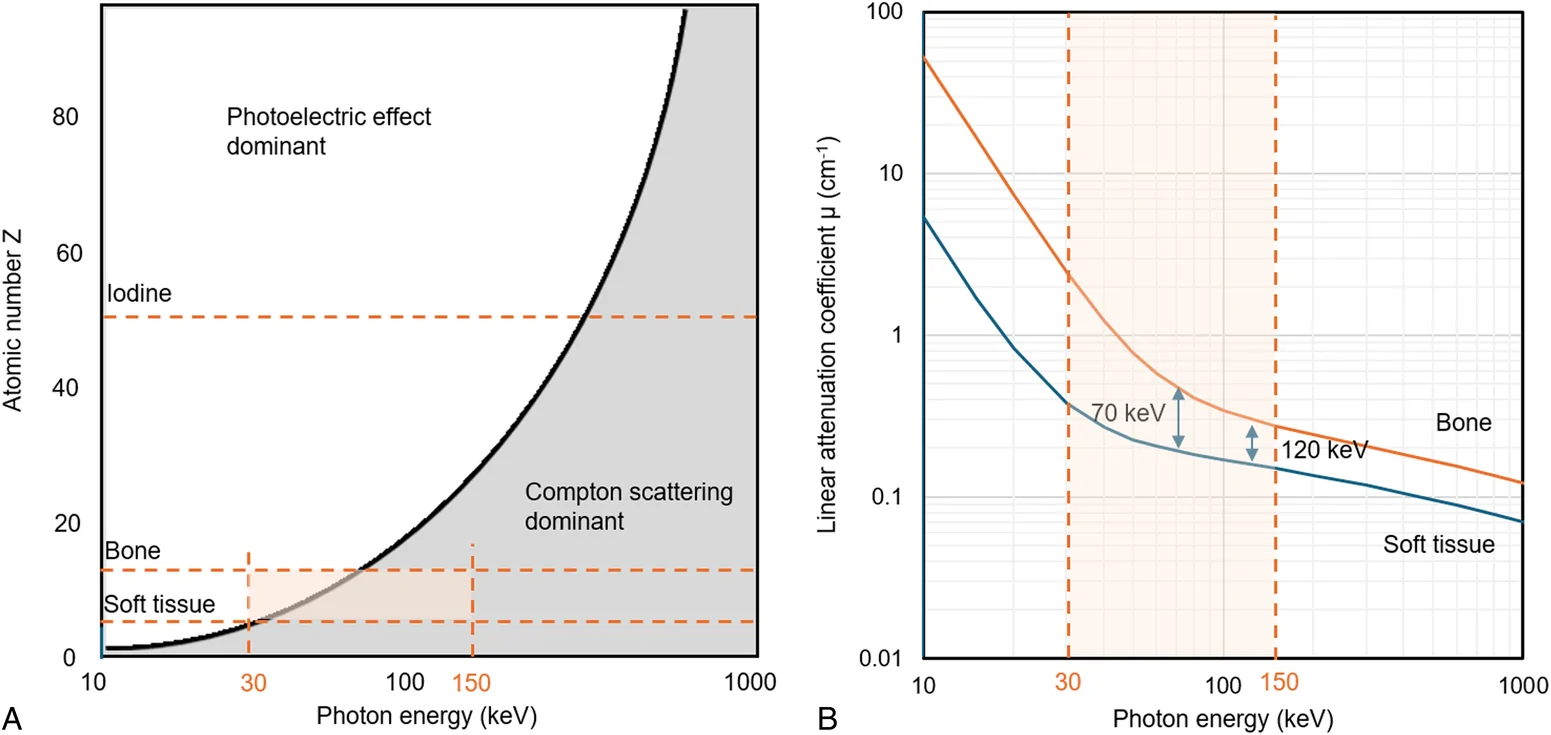
A, Graph illustrating the relative importance of the photoelectric effect and Compton scattering for different atomic numbers (linear scale) and photon energies (logarithmic scale). B, Graph showing the linear attenuation coefficient of bone and soft tissue (logarithmic scale) plotted against the photon energy (logarithmic scale). The orange areas in both graphs indicate the relevant CT energy range. The blue arrows denote the difference between bone and soft tissue at 70 and 120 keV, which is related to the contrast between the two tissues.^10^

Thus, image contrast depends on both the x-ray spectrum energy and tissue composition.^6^ In general, attenuation decreases as photon energy increases, because higher-energy photons are less likely to interact with matter. Figure 2B illustrates this for bone and soft tissue: while bone shows much stronger attenuation than soft tissue at low energies, the curves converge at higher energies where Compton scattering dominates.^10^ Consequently, bone-soft tissue contrast diminishes at higher energies, as their attenuation becomes more similar.

Noise in CT refers to random pixel intensity fluctuations that can obscure fine detail and reduce diagnostic confidence.^8^ It arises from quantum noise (due to statistical variation in the number of photons reaching the detector) and electronic noise (unwanted electrical signals from the CT electronics).^6^ Electronic noise becomes particularly noticeable when photon counts are low, typically due to high attenuation (e.g., obese patients or dense tissues) or low tube current (mAs). Increasing photon energy (higher kV) improves penetration and thus reduces noise, at the cost of image contrast. Conversely, lowering kV increases contrast but also increases noise at the same radiation dose. The contrast increase is particularly pronounced for iodine and can outweigh the noise increase. Therefore, modern CT scanners incorporate tube voltage selection algorithms that propose an appropriate kV based on patient size and clinical indication to optimize contrast, noise, and radiation dose.

### Conventional Computed Tomography Technology

Conventional CT scanners use EIDs that convert absorbed x-ray photons into visible light through a scintillator, subsequently detected by photodiodes (so-called indirect detection). EIDs measure total energy over time and cannot distinguish individual photon energies, causing loss of (spectral) information. Consequently, in conventional CT using a single kV, materials with similar attenuation for that kV value yield similar CT values in a CT-image and cannot be differentiated.^11–13^

### Dual Energy Computed Tomography Technology

Because photoelectric effect and Compton scattering depend differently on photon energy, material attenuation varies more at low energies and converges at higher energies (Fig. 2B). DECT uses this characteristic by acquiring data at 2 distinct effective energies, allowing comparison of attenuation behavior between materials.^2^

By analyzing energy-dependent attenuation, DECT can distinguish 2 materials (see Table 2 for the underlying mathematics of material decomposition). For instance, soft tissue and iodine can both have similar low CT values at a high mean energy. At low effective energy, iodine—due to its higher atomic number and thus more photoelectric effect—shows significantly increased attenuation, enabling effective material separation.^3,16^ This allows the generation of material-specific maps and virtual noncontrast images by subtracting iodine contributions.

**TABLE 2:**
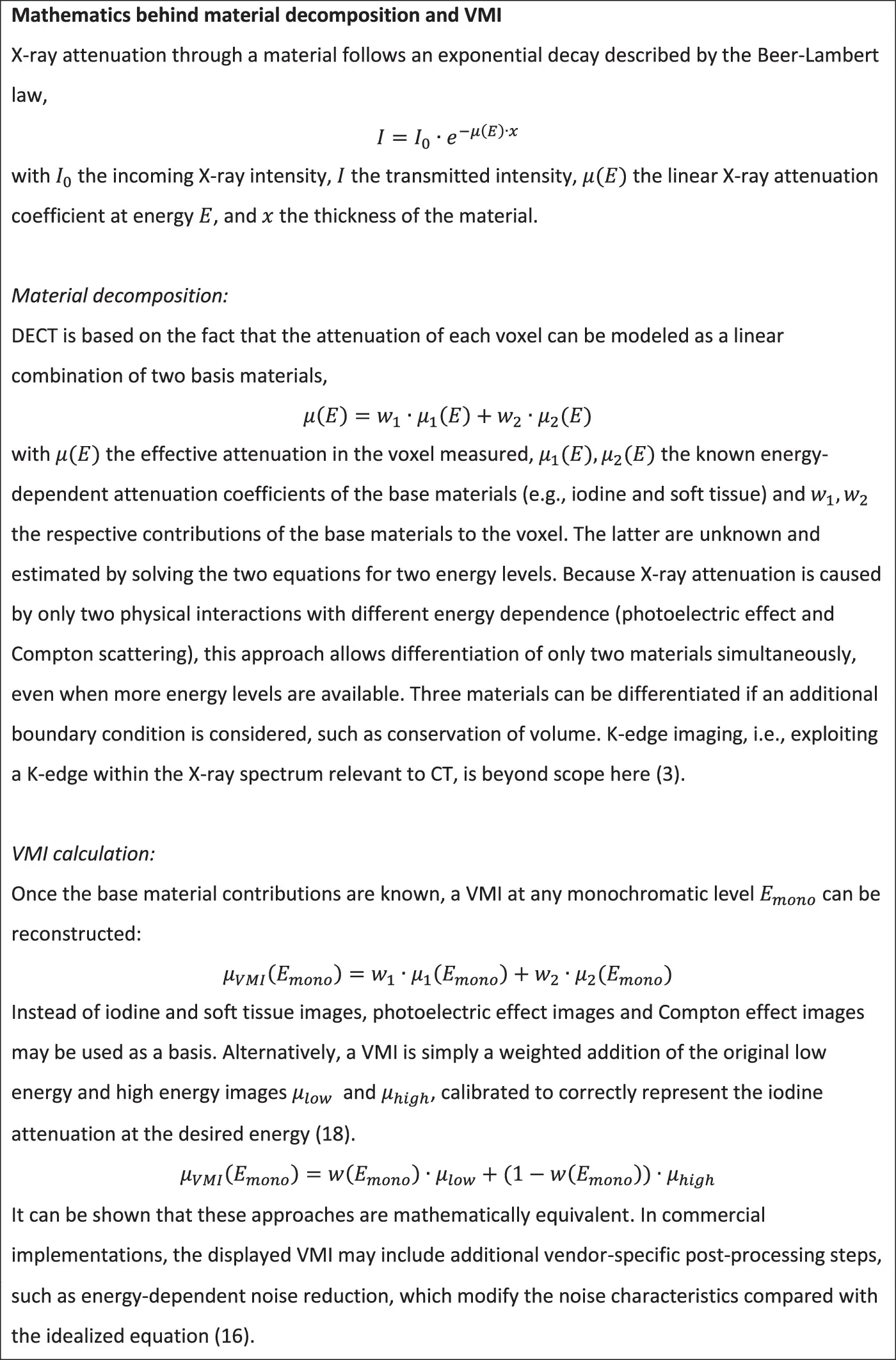
Technical Note (Optional Read)

Another impactful post-processing application of DECT is VMIs.^14,15,17,18^ VMIs are reconstructed from spectral CT data to simulate how each image voxel would appear if scanned with a single photon energy (eg, 70 keV), rather than with a polyenergetic spectrum (eg, 120 kV). Users can select a specific VMI energy during image reconstruction, optimized for the clinical task. In this way, the prospective, kV-based determination of image characteristics is replaced by a retrospective keV-adjustment. Still, the optimal acquisition kV must be determined dependent on patient size and tube power requirements.^19^ However, depending on the CT-system, the selection may be limited to fewer options (eg, 120 kV or 140 kV for PCD-CT in spectral standard mode).

VMIs (particularly at higher keV, ∼≥70 keV) can mitigate beam-heardening artifacts and provide more predictable attenuation values.^20–22^ Moreover, VMIs allow for the reconstruction of low keV images, which increase iodine contrast but also increase noise. In addition, high keV images can be reconstructed, which reduces noise and can partially reduce metal artifacts, although they cannot fully replace dedicated metal artifact reduction techniques.^23^ Importantly, in commercial implementations, the displayed VMI may include additional vendor-specific post-processing steps, such as energy-dependent noise reduction, which modify the noise characteristics compared with the idealized equation.^15^

### Photon-counting Computed Tomography Technology

PCDs convert photons directly into electrical signals, enabling measurement of each individual photon’s energy (Fig. 3). Each photon produces a voltage peak proportional to its energy. By applying an energy threshold, (low-energy) electronic noise can be eliminated, improving IQ. Detected photons are read-out into energy bins, inherently enabling multienergy data acquisition from a single x-ray spectrum. This enables reconstruction of VMIs from a single acquisition.^12,24^ Moreover, PCD-CT enhances the contribution of low-energy photons because each photon is counted equally regardless of its energy. In contrast, in EID-CT, high-energy photons contribute more to the summed (energy-integrated) signal than the low-energy photons. Since low-energy photons carry valuable contrast information, their enhanced contribution in PCD-CT improves the quality of VMIs with respect to VMIs calculated with DE.

**FIGURE 3:**
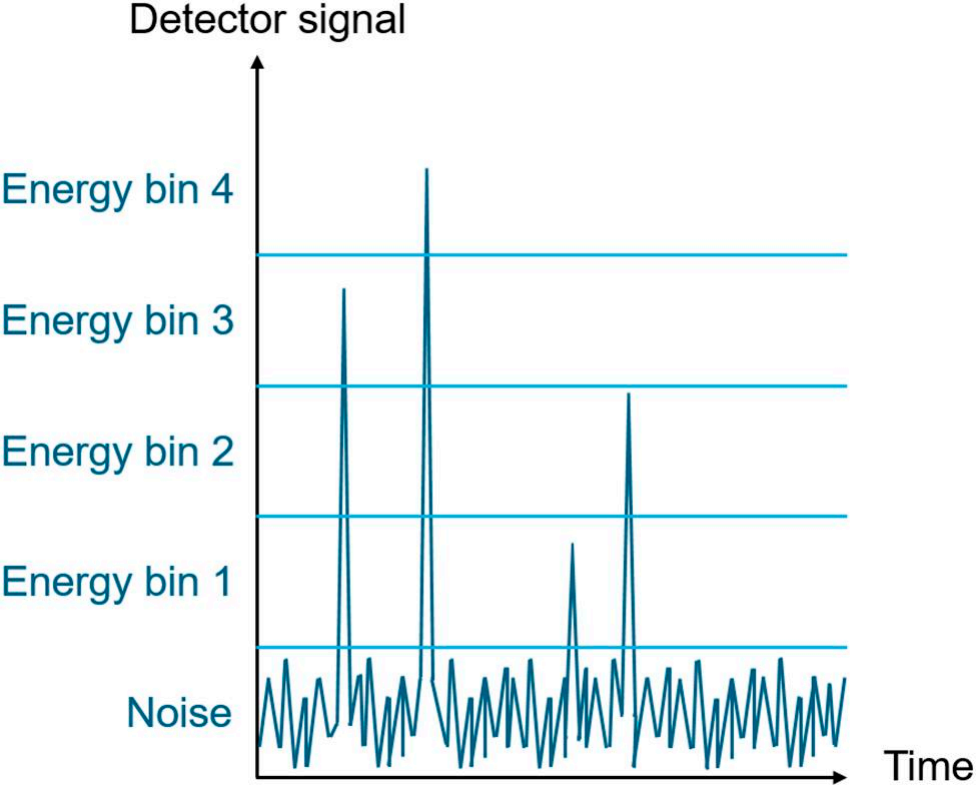
Graph illustrating the signal detected by a photon-counting detector, which is able to register individual photon energy peaks and to sort them in their corresponding energy bin.

It is important to note that in PCD-CT, spectral data sets are available by default. This shifts the focus from polyenergetic CT images to standard viewing of VMIs at a chosen keV-level (Table 3). Nevertheless, kV selection remains relevant, as it defines the upper energy limit of the x-ray beam and affects penetration as well as the available tube power reserves. Whereas for polyenergetic images, often lower kV is used to optimize contrast, in PCD-CT, the focus is more on ensuring sufficient spectral information across the energy range, which favors higher kV. Lower kV, often used for dose reduction in pediatrics, narrows the available energy spectrum for VMI reconstruction.

**TABLE 3:** Key Concept

VMI reconstructions at a certain keV-level (either with DECT or PCD-CT) are NOT equivalent to conventional kV images. VMIs SIMULATE images as if acquired with monoenergetic photon beams based on mathematical modeling of attenuation and material decomposition. Conversely, conventional kV images are acquired using a polychromatic spectrum, where the detector integrates the total deposited energy. As a result, attenuation values, contrast behavior, and noise characteristics in VMIs differ from those in kV images, and they cannot be directly matched or substituted one-to-one. However, for each kV setting on EID-CT, the corresponding mean photon energy of the polychromatic x-ray spectrum can be calculated using a validated spectrum simulation tool (e.g., SpekPy). Although the mean energy of a kV spectrum is not identical to the respective VMI keV-level, this approach provides a reasonable approximation for matching the energy content of the polyenergetic spectra to the VMI keV-level.

## PHANTOM SET-UP AND METHODOLOGY

To assess IQ for polyenergetic images and VMIs across varying kV and keV settings, an anthropomorphic phantom (Kyoto PBU-60, Kyoto Kagaku Co, Ltd) was scanned with 9 material Gammex rods (Sun Nuclear – a Mirion Medical Company; Fig. 4A). The rods were placed on the anterior side of the Kyoto phantom, directly above its liver region. These included 7 iodine concentrations (2.0 to 20.0 mg/mL), covering a clinically relevant range, as well as liver and muscle-equivalent rods, as defined by the International Commission on Radiation Units and Measurements (ICRU).^25^ Scans were performed on a third-generation dual-source EID-CT (SOMATOM Force, Siemens) and on a first-generation dual-source PCD-CT (NAEOTOM Alpha, Siemens Healthineers), with the phantom centered at the isocenter of both scanners.

**FIGURE 4:**
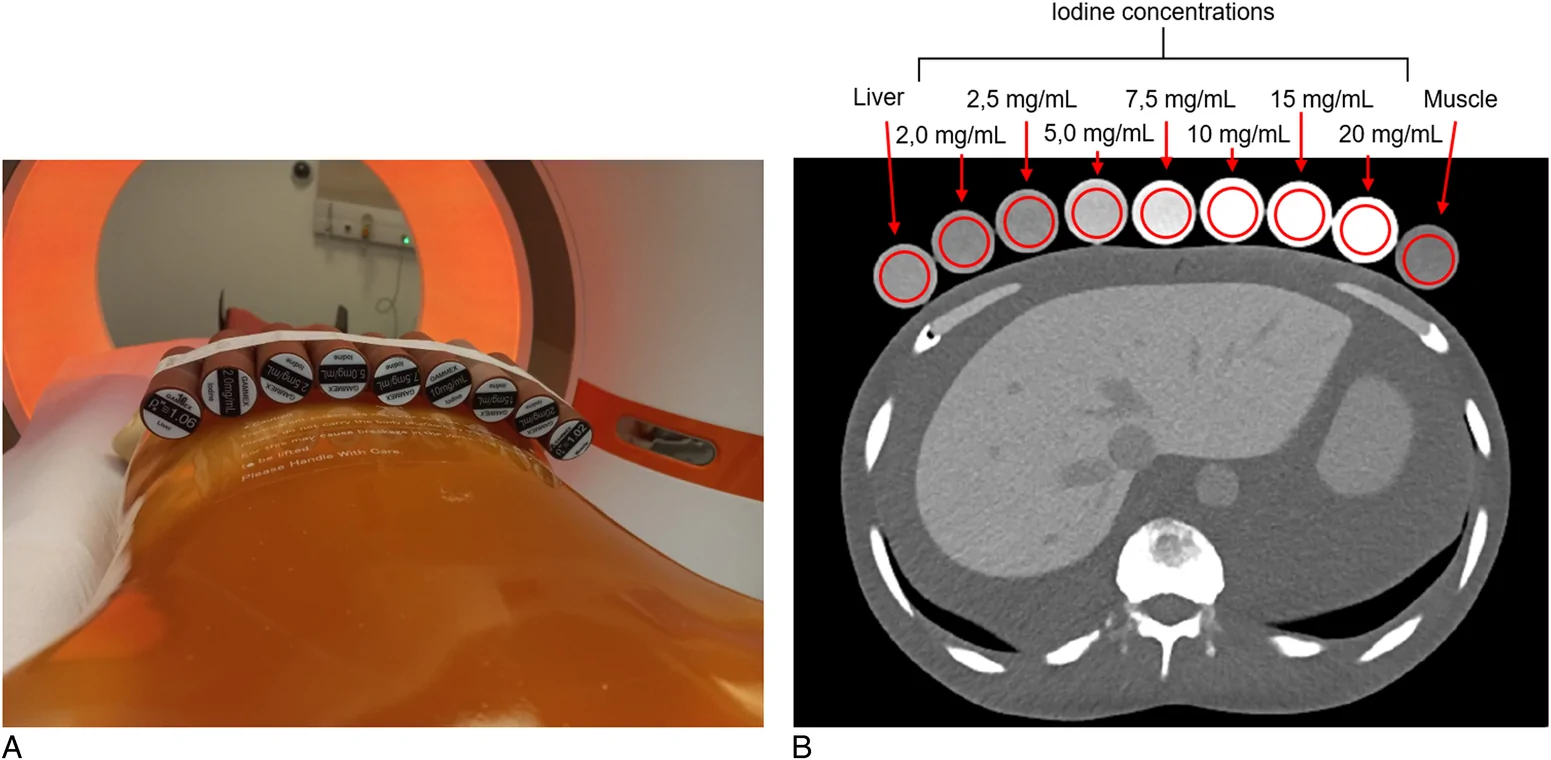
A, Photo of the setup of the Kyoto phantom with the Gammex rods. B, Example scan (window width 400, window level 40) of the phantom set-up with circular regions of interest (ROIs) drawn in the iodine rods, liver rod, and muscle rod of the Kyoto phantom. These ROIs were used for measurements of mean attenuation, contrast, and contrast-to-noise ratio.

Detailed acquisition and reconstruction parameters are provided in Table 4a. Although a dual-source CT system was used, polyenergetic images on the EID-CT were acquired in conventional single-kV mode, at tube voltages from 70 to 140 kV, in 10 kV steps. On the PCD-CT, data were acquired at a fixed 120 kV tube voltage (clinical abdominal protocol). VMIs were reconstructed in a range of 53 to 90 keV, at keV levels matching with the effective energies of the EID-CT spectra according to Table 4b, which were obtained from measurements of the attenuation of a 15 mg/mL iodine rod in a 20 cm water phantom and comparison to NIST tables of the expected keV-dependent attenuation. The effective energy as received by the detector depends on the size of the phantom. As the Kyoto phantom is larger than the 20 cm water phantom used for Table 4b, the measured kV-dependent CT-values of the EID-CT were eventually matched to the corresponding CT-values of the VMIs at the respective keVs to get an approximation of the effective energies of the EID-CT spectra with the Kyoto phantom. This enables a direct comparison of the corresponding noise and CNR results. In addition, a polychromatic image (T3D image) was made, reconstructed from the low-energy threshold PCD-CT data, for which the CT values were also matched to the CT-values of the EID-CT to obtain the effective energy. Moreover, for both scanners, images were acquired across a range of CT Dose Index volume (CTDI_vol_) values (2 to 25 mGy). Scans were reconstructed with iterative reconstruction (ADMIRE 3 on the EID-CT, QIR 3 on the PCD-CT) with equivalent soft tissue kernels.

**TABLE 4:** Overview of Acquisition and Reconstruction Parameters

CT Parameters	Third-generation Dual-source EID-CT	First-generation Dual-source PCD-CT
a)		
Peak tube voltage	70-140 kV†	120 kV (reconstructed for 53-90 keV)†
CTDI_vol_ (mGy)	2-25*	2-25
Slice thickness (mm)	3	3
Increment (mm)	2	2
Pitch	0.9	0.9
Acquisition mode	Axial	Axial
Gantry rotation time (s)	0.5	0.5
FoV (mm)	320	320
Iterative reconstruction	ADMIRE 3	QIR 3
Kernel	Br40d	Br40
b)		
Polyenergetic x-ray spectrum (kV)	Approximate effective energy (keV) for a 15 mg/mL iodine rod in a 20 cm water phantom	
70	53	
80	57	
90	61	
100	65	
110	66	
120	67	
130	69	
140	70	

* Due to tube power limitations, some high CTDI_vol_ values cannot be achieved at low kV settings on the EID-CT.

† See Table 4b for the actual kV and keV values used. Table 4b gives an approximation of the effective energy of the kV spectra on the third-generation dual-source EID-CT in keV, which were obtained from measurements of the attenuation of a 15 mg/mL iodine rod in a 20 cm water phantom and comparison to NIST-tables of the expected keV-dependent attenuation.

EID-CT, energy-integrating detector computed tomography; PCD-CT, photon-counting detector computed tomography; CTDI_vol_, computed tomography dose index volume; FoV, field of view.

### Image Quality Parameters

Image quality measurements were conducted on a single axial slice corresponding to the largest visible liver cross-section. Mean attenuation was measured in all iodine, liver, and muscle rods of the phantom by placing circular ROIs with 1 cm radius centered in the rods (Fig. 4B). Contrast was calculated as the signal difference between the ROIs and the muscle ROI. In addition, the global noise level (GNL) was determined by calculating the SD of pixel values using a 7×7 mask centered on pixels within the soft tissue attenuation range.^26^ A histogram of these SDs was generated, and the GNL was defined as the median SD, providing a measure of image noise in homogeneous soft tissue regions. Lastly, CNR was calculated as contrast divided by the GNL.

## INFLUENCE OF ENERGY SELECTION IN COMPUTED TOMOGRAPHY ON IMAGE QUALITY

### Effect of kV/keV on Contrast

Figure 5A compares mean attenuation values of iodine (5 and 10 mg I/mL), liver, and muscle rods obtained with polyenergetic images from EID-CT and PCD-CT VMIs. Results for the other concentrations are provided in the Supplementary Material (Supplemental Digital Content 1, http://links.lww.com/RLI/B108). For the polyenergetic images, attenuation is shown versus polyenergetic tube voltage (kV), and for the VMIs from the PCD-CT as a function of the reconstructed energies (keV). For 10 mg I/mL, attenuation decreases with increasing energy for both imaging types, from ~420 HU at 70 kV to ~180 HU at 140 kV in polyenergetic EID-CT images, and from ~460 HU at 53 keV to ~150 HU at 90 keV in the VMIs. The measured kV-dependent CT-values of the EID-CT are matched to the corresponding CT-values of the VMIs at the respective keVs to get an approximation of the effective energies of the spectra (Table 1 in the Supplementary Material, Supplemental Digital Content 1, http://links.lww.com/RLI/B108).

**FIGURE 5:**
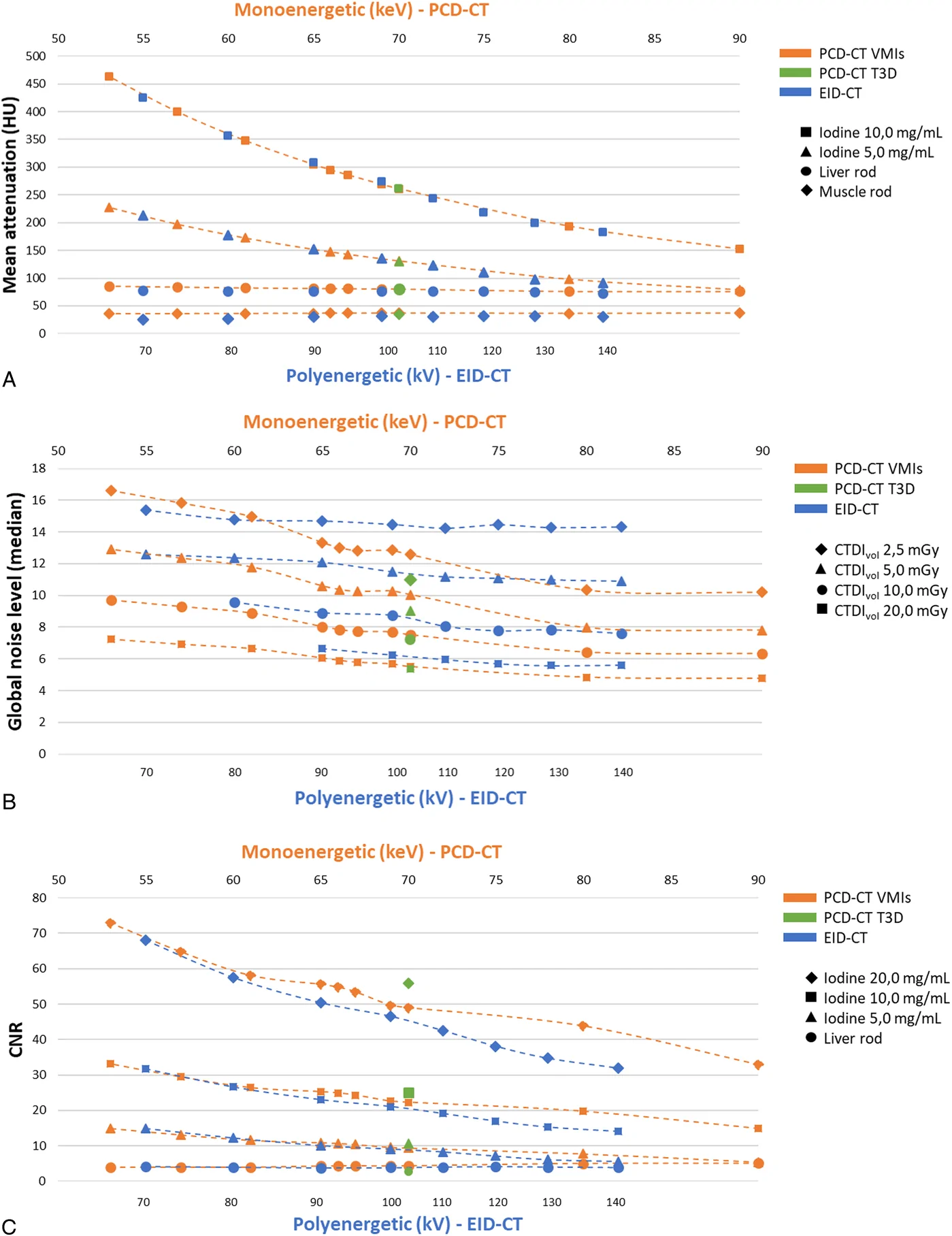
A, Mean attenuation values (in Hounsfield Units; HU) measured in the iodine (5.0 mg/mL and 10.0 mg/mL), liver, and muscle rods across different energy levels for CTDI_vol_=5 mGy. B, Global noise level (GNL) measured for different CTDI_vol_ levels across different energy levels. C, Contrast-to-noise ratio (CNR) measured in different iodine rods and a liver rod across different energy levels for CTDI_vol_=5 mGy. Results are shown for both polyenergetic images from an energy-integrating detector CT (EID-CT, lower *x*-axis), and virtual monoenergetic images and a T3D reconstruction from a photon-counting detector CT (PCD-CT, upper *x*-axis). Note that the effective energy of the kV values on the lower x-axis approximately corresponds to keV levels on the upper *x*-axis; as a result, the lower x-axis does not follow a linear scale.

The mean CT values for the liver and muscle rods remain relatively constant across this energy range, ~80 HU for liver and ~40 HU for muscle, because attenuation in low-Z tissues is dominated by the Compton effect, which changes only slightly with photon energy. Liver exhibits slightly higher CT values than muscle due to its higher electron density and, consequently, a higher probability of Compton scattering. Furthermore, as PCD-CT is less affected by beam hardening, the effective energy of the T3D is lower compared with the 120 kV polyenergetic EID-CT scan.

Since iodine attenuation decreases with increasing photon energy (due to diminishing photoelectric effect), whereas muscle attenuation remains nearly constant (Compton-dominated), image contrast (Section 'Image contrast and noise in Computed Tomography') decreases at higher energies. As expected, the 10 mg I/mL rod demonstrates higher attenuation, and therefore higher contrast, than the 5 mg I/mL rod for both scanner types, with clear separation between the concentration levels across the full energy range. Higher iodine concentrations yield steeper attenuation curves and thus more contrast, because a larger amount of iodine attenuated more x-rays, particularly at lower energies.

Figure 6 shows clinical example scans of the same oncology patient, acquired with EID-CT at 90 kV (top) and with a PCD-CT (bottom). The VMIs were reconstructed at 65 keV, corresponding to the effective energy of the polyenergetic acquisition. In addition, the intrinsically available spectral information of the PCD-CT allowed for reconstruction at lower and higher keV. Reconstructions at 55 and 82 keV are shown, demonstrating the enhanced and reduced contrast, respectively.

**FIGURE 6:**
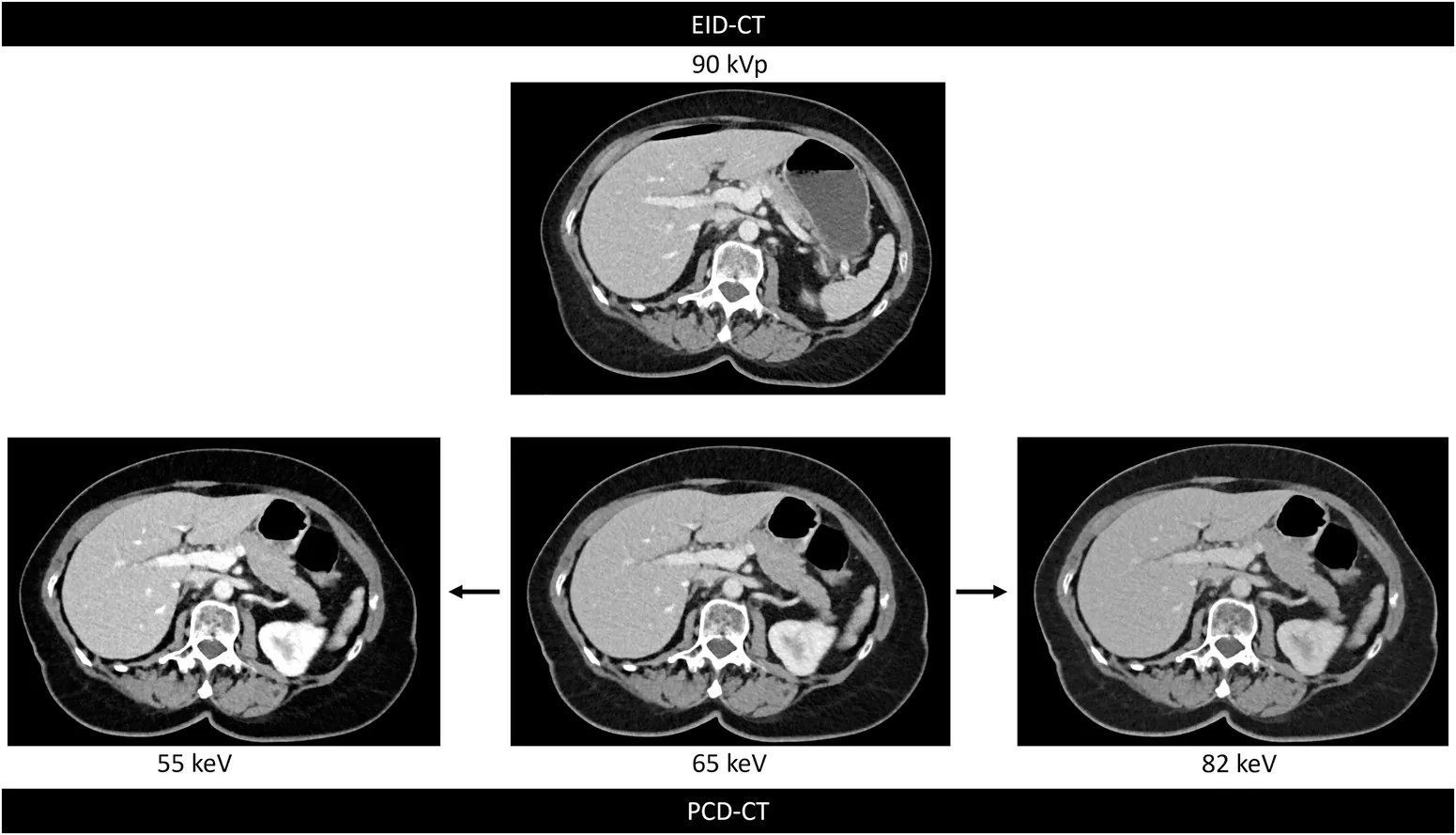
Clinical example scan of an oncology patient (female, 77y, BMI 21.9 kg/m^2^) made with an EID-CT (upper row, 90 kV) and a follow-up scan 5 months later of the same patient (BMI: 22.4 kg/m^2^) with a PCD-CT (lower row). The PCD-CT scan was acquired with 120 kV and reconstructed at 65 keV, corresponding to the effective energy of the 90 kV x-ray spectrum of the used EID-CT. In addition, the PCD-CT images are reconstructed at 55 keV (corresponding to ~70 kV) and 82 keV (corresponding to ~140 kV) to demonstrate the intrinsically available spectral information, showing enhanced and reduced contrast, respectively (the clinical images shown in this manuscript are included for illustrative purpose only. They were acquired as part of routine clinical care and were retrospectively selected. Ethics committee approval was waived by the institutional review board).

### Effect of kV/keV on Noise

Figure 5B shows the GNL as a function of energy for both polyenergetic EID-CT images and PCD-CT VMIs at different CTDI_vol_ levels. For both detector types, noise was lower for higher radiation dose, as expected due to the larger number of detected photons resulting in reduced statistical variation (ie, improved photon statistics). At the lower CDTI_vol_ level of 2.5 mGy, noise would theoretically double compared with 10 mGy scans (as noise ∝ √dose). However, the actual noise increase observed is only a factor of 1.6 to 1.8. This may be caused by an increased post-processing data filtration at low radiation dose levels to reduce potential noise streaks.

For the polyenergetic images, the GNL at a given CTDI_vol_ remained relatively stable across the energy range, with a slight noise reduction at higher kV values due to improved photon penetration, resulting in more photons reaching the detector. In the VMIs, the GNL showed a more pronounced decrease at higher energy levels, especially for lower radiation doses. In VMIs of PCD-CT, noise should theoretically increase significantly the further the keV values deviate from the effective energy of the spectrum of the CT scan (here 120 kV, corresponding to 75 keV). Theoretically, the VMI curves shown in Figure 5B should have a minimum at about 75 keV, and the noise should rise substantially, especially for lower keVs. This is a consequence of the mathematical combination of low- and high-energy data when reconstructing VMIs. To obtain low-energy VMIs from a higher-energy CT scan (120 kV), high weighting factors (see Table 2) must be applied, which amplify image noise. However, modern image processing in VMI reconstruction prevents excessive noise at both ends of the energy spectrum.^15^ It is fine-tuned, resulting in the noise curves shown in Figure 5B. While noise in polyenergetic images is a natural consequence of the underlying physical principles, the energy dependence of noise in VMIs is strongly determined by image processing.^15^

The noise in the polychromatic (T3D) image of the PCD-CT at 120 kV (effective energy 70 keV) is lower than the noise in the 120 kV image of the EID-CT (effective energy 75 keV), especially at lower CTDI_vol_. One reason for this may be the absence of electronic noise in PCD-CT.

Figure 5C shows the CNR values for multiple iodine concentrations at a fixed radiation dose across different energy levels for both polyenergetic images and VMIs. For both imaging types, CNR decreases with increasing energy due to the reduction in iodine contrast at higher energies. However, for polyenergetic images, the decline in CNR at higher energies is more pronounced than for the VMIs. This is a result of the noise pattern observed in Figure 5B. Iodine CNR in VMIs is similar to polyenergetic EID-CT images for energies up to 60 keV, and higher for higher energies.

## SUMMARY AND CONCLUSIONS

This review illustrates the fundamental difference between conventional tube potential (kV) in polyenergetic CT images from an EID-CT and VMIs (keV) from a PCD-CT. While both parameters describe the “energy” of the x-ray beam, they are conceptually different. A kV setting defines a polyenergetic x-ray spectrum with a broad range of photon energies, whereas keV levels represent VMIs, simulating images as if acquired with a single photon energy.

This review does not aim to provide a head-to-head performance comparison between EID-CT and PCD-CT, but rather to illustrate how fundamental differences in image formation between polyenergetic and monoenergetic imaging with PCD-CT affect contrast and noise characteristics, even at comparable energy levels. In this work, VMIs were reconstructed from PCD-CT data, but it should be emphasized that VMIs can also be generated with other spectral CT technologies. Likewise, polyenergetic images can be generated from PCD-CT data.

Understanding the differences is essential for interpreting attenuation and noise behavior. In polyenergetic images from an EID-CT, increasing the kV hardens the x-ray beam, shifts the effective energy upward, and thereby reduces iodine contrast. Increasing the keV level in PCD-CT VMIs reduces contrast as well. Regarding noise, both systems show reduced noise at higher radiation doses, but PCD-CT demonstrates a stronger energy dependence with a reduction in noise for higher energy levels. CNR is similar for energies up to 60 keV in the VMIs from the PCD-CT and in the polyenergetic EID-CT images, and higher in the VMIs at higher energies because it decreases less rapidly. Clinically, this allows for retrospective optimization of IQ without needing multiple acquisitions at different tube voltages.^27^ This may improve diagnostic confidence, particularly when assessing iodine-enhanced lesions. Moreover, the ability to select optimal VMI levels post-acquisition may facilitate the optimization of acquisition protocols in future examinations, which could contribute to radiation dose reduction while maintaining diagnostic IQ. In clinical practice, automated VMI optimization tools such as CARE keV already prospectively link radiation dose to a task-specific VMI energy.^19^ In that context, dose optimization has already been performed before acquisition, and the benefit of retrospective VMI adjustment may be limited to fine-tuning image impression, enhancing contrast in the event of a missed contrast bolus, or reducing metal artifacts.

In the presented phantom experiment, kV spectra were linked to VMI keV levels by matching the effective photon energy of the polyenergetic beam. Although this is not identical to a true monoenergetic acquisition, it provides a reasonable and intuitive framework for comparison. Although the quantitative results of this study are specific to the scanners used, the trends observed are likely generalizable to other scanners. Absolute values may differ due to variations in detector technology, reconstruction algorithms, and filtration.

This review is limited by the fact that both scanners are from a single vendor, as only one commercial PCD-CT system was available at the time. However, the educational insights regarding polyenergetic versus monoenergetic imaging are physics-driven, and the essence will be the same for different vendors. Also, the present study focused on image quality in terms of contrast and noise; other factors such as spatial resolution were not evaluated. Reported improvements in spatial resolution for PCD-CT are primarily related to intrinsic detector properties rather than to virtual monoenergetic reconstruction.^4^ Furthermore, while this review focused on iodine-based soft-tissue contrast, other applications such as calcium discrimination or lung nodule imaging may exhibit different spectral characteristics and optimal energy ranges, which should be explored in future studies.

In conclusion, kV and keV should not be seen as interchangeable terms. kV defines the x-ray spectrum at acquisition, whereas keV in VMIs is a reconstruction parameter and not a direct modulation of beam energy. Both determine the trade-off between image contrast and noise, but PCD-CT and DECT uniquely offer spectral data that allow radiologists to optimize IQ retrospectively by selecting the most suitable keV levels.

## Supplementary Material
