# Supplementary material for: It Is All About Energy-the Changing Role of X-ray Photons in Photon-counting Detector Computed Tomography: What the Radiologist Needs to Know About kV and keV
Source: Invest Radiol. 2026 Apr 6;61(11):797–805. doi: 10.1097/RLI.0000000000001283 (PMC13629652; doi:10.1097/RLI.0000000000001283)
Supplement: Supplementary file 1 [file rli-61-797-s001.pdf]

Supplementary material

Table 1 *Approximation of the effective energy of the kV spectra on the dual-source EID-CT in keV experimentally obtained for the Kyoto phantom.*

Polyenergetic X-ray spectrum (kV)	Approximate effective energy (keV) for the Kyoto phantom
70	55
80	60
90	65
100	69
110	72
120	75
130	78
140	82

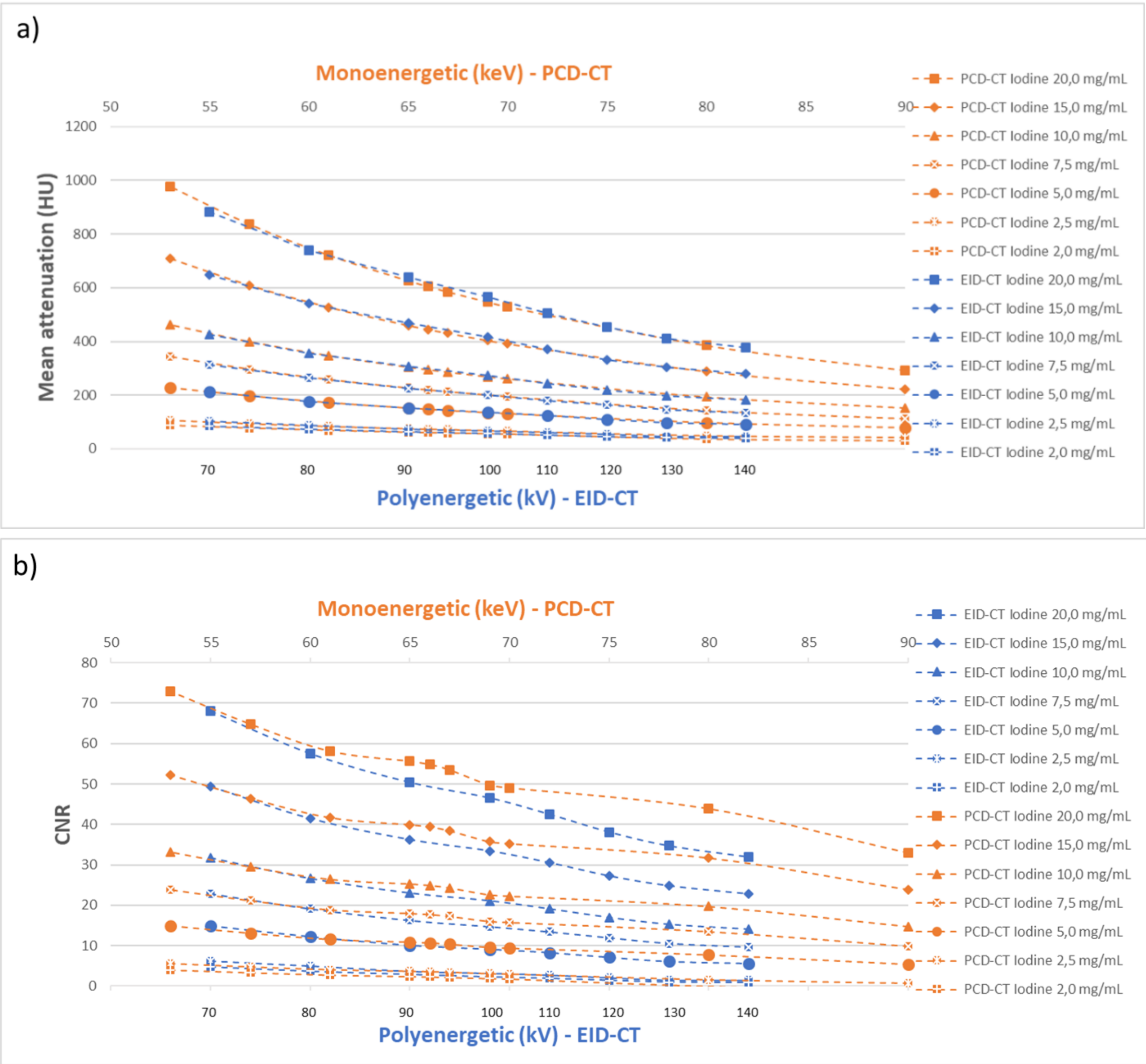

***Figure 1** a) Mean attenuation values (in Hounsfield Units; HU) and b) Contrast-to-noise ratio (CNR). Results are measured in rods with varying iodine concentrations across different energy levels for CTDIvol = 5 mGy and shown for both polyenergetic images from an energy-integrating detector CT (EID-CT, lower x-axis), and virtual monoenergetic images from a photon-counting detector CT (PCD-CT, upper x-axis). Note that the effective energy of the kVp values on the lower x-axis approximately corresponds to keV levels on the upper x-axis; as a result, the lower x-axis does not follow a linear scale.*
